# Anti-Inflammatory Properties of Yellow Passion Fruit Bagasse Extract and Its Potential Role in Periodontal Wound Healing In Vitro

**DOI:** 10.3390/biomedicines13051134

**Published:** 2025-05-07

**Authors:** Andressa V. B. Nogueira, Luan V. Faria, Maria Eduarda S. Lopes, Juliane Viganó, Julian Martínez, Sigrun Eick, Joni A. Cirelli, James Deschner

**Affiliations:** 1 Department of Periodontology and Operative Dentistry, University Medical Center of the Johannes Gutenberg University, 55131 Mainz, Germany; lv.faria@unesp.br (L.V.F.); maria.e.lopes@unesp.br (M.E.S.L.); james.deschner@uni-mainz.de (J.D.); 2 Department of Diagnosis and Surgery, School of Dentistry at Araraquara, São Paulo State University—UNESP, Araraquara 14801-385, SP, Brazil; joni.cirelli@unesp.br; 3 Faculdade de Zootecnia e Engenharia de Alimentos (FZEA), Universidade de São Paulo (USP), Pirassununga 13635-900, SP, Brazil; jvigano@usp.br; 4 Faculty of Food Engineering, Department of Food Engineering, University of Campinas (UNICAMP), Campinas 13083-862, SP, Brazil; julian@unicamp.br; 5 Department of Periodontology, School of Dental Medicine, University of Bern, 3010 Bern, Switzerland; sigrun.eick@unibe.ch

**Keywords:** periodontal ligament cells, *Passiflora edulis* sp. Bagasse, *Fusobacterium nucleatum*, periodontitis, inflammation

## Abstract

**Background/Objectives**: Periodontal disease involves chronic immunoinflammatory processes and microbial dysbiosis, making phytochemicals with anti-inflammatory properties potential therapeutic agents. This study aimed to assess the modulatory effects of yellow passion fruit bagasse extract (PFBE) on periodontal cells under microbial condition. **Methods**: A human periodontal ligament (PDL) cell line was exposed to *F. nucleatum* ATCC 25586 to simulate a microbial environment in vitro in the presence and absence of PFBE containing three different concentrations (0.25, 0.50, and 1.00 µg/mL) of piceatannol. Pro-inflammatory markers (TNF-α, IL-8, CCL2), the antioxidant enzyme SOD2, and the protease marker MMP-1 were analyzed by real-time PCR. Protein levels were assessed via ELISA and NF-κB nuclear translocation by immunofluorescence. Cell viability was investigated using live/dead and alamarBlue assays, and in vitro wound healing was evaluated by an automated scratch assay. **Results**: PDL cells exposed to *F. nucleatum* significantly increased the gene and protein expression of all inflammatory markers. The stimulatory effects of *F. nucleatum* were significantly reduced when PDL cells were simultaneously exposed to PFBE. *F. nucleatum* triggered the NF-κB nuclear translocation while PFBE abrogated the *F. nucleatum*-stimulated NF-κB nuclear translocation at 60 min. Viability assays demonstrated that neither PFBE nor *F. nucleatum* were toxic or significantly affected PDL cell viability. In vitro wound closure was improved by the addition of PFBE to *F. nucleatum*. **Conclusions**: PFBE exhibited anti-inflammatory and anti-proteolytic effects while improving in vitro wound healing, suggesting a potential modulatory role of PFBE in periodontal disease prevention and treatment.

## 1. Introduction

Periodontitis is a chronic inflammatory disease characterized by the progressive destruction of the tooth-supporting structures, ultimately leading to tooth loss if left untreated. Its occurrence and progression are driven by microbial dysbiosis associated with an imbalance in immune homeostasis [1]. Evidence confirms that periodontitis is influenced by multiple risk factors, such as smoking, inadequate oral hygiene, genetic and epigenetic predispositions, and stress. Furthermore, periodontitis is strongly associated with various systemic diseases and conditions, including diabetes mellitus, obesity, metabolic syndrome, and cardiovascular diseases [2,3]. Given its high global prevalence, periodontitis represents a significant public health burden, not only impairing patients’ quality of life but also imposing substantial socioeconomic costs [4,5].

The primary periodontal treatment approach involves thorough mechanical removal of the periodontal biofilm by subgingival instrumentation, coupled with optimal oral hygiene practices [6]. This strategy has demonstrated efficacy in reducing periodontal inflammation and improving clinical outcomes [7]. In most cases, however, this treatment does not result in a complete healing of the disease but only in a retardation of the disease progression. Recurrence and tooth loss are still common sequela [8]. In addition, even if the treatment of this inflammatory disease has been successful, there may still be esthetic problems, dentinal hypersensitivity, and a higher risk of root caries. Consequently, there is an increasing interest in developing and integrating adjunctive therapeutic modalities to enhance the prevention and management of periodontal diseases [6].

Phytochemicals are bioactive chemical compounds naturally present in fruits and vegetables that have been extensively studied for their pharmacological properties [9]. Among phytochemicals, polyphenols are particularly notable for their potential health benefits, such as antioxidant, antimicrobial, anti-inflammatory, antiallergic, anticancer, and antihypertensive activities [10,11,12,13]. Within the field of oral health, polyphenols have shown significant beneficial properties. In the context of periodontal diseases, they exert anti-inflammatory and immunomodulatory effects by reducing the pro-inflammatory markers involved in periodontal tissue destruction while increasing anti-inflammatory mediators [10,13,14]. In addition, polyphenols exhibit an antimicrobial capacity against key periodontal pathogens, such as *Porphyromonas gingivalis*, *Aggregatibacter actinomycetemcomitans*, and *Fusobacterium nucleatum* [15,16]. *F. nucleatum*, for example, is a Gram-negative anaerobe that is very common on the dorsal surface of the tongue and is an important component of the multi-species biofilms present on the gingival margin, thus playing a major role in the plaque formation. Furthermore, it is a central pathogen in periodontal infections [17]. Beyond the anti-inflammatory and antimicrobial properties, polyphenols reduce oxidative stress through their antioxidant activity [18]. They may promote tissue regeneration and wound healing, which could be particularly valuable in cases of periodontal tissue loss. Given these multifaceted benefits, incorporating bioactive compounds into periodontal treatment strategies presents a promising possibility for improving clinical outcomes, especially in cases where conventional therapies are hindered by irreversible tissue destruction or other complications.

Yellow passion fruit (*Passiflora edulis* Sims) is a rich source of bioactive compounds, mainly polyphenols [19]. Among these, piceatannol, a hydroxylated derivative of resveratrol, is the predominant polyphenol in passion fruit seeds [20]. Notably, a catechol group in its B-ring confers piceatannol with enhanced antioxidant capacity compared to resveratrol [21]. In addition to its antioxidant activity, piceatannol has distinguished anti-inflammatory and antitumor effects [22,23,24]. Piceatannol extract derived from passion fruit seeds has demonstrated antioxidant capacity in periodontal ligament (PDL) cells under oxidative stress conditions [25].

Furthermore, polyphenols from *Passiflora edulis* can modulate inflammatory pathways by inhibiting pro-inflammatory cytokines while reducing oxidative stress markers [26]. Moreover, the antimicrobial properties of passion fruit extracts further contribute to controlling the growth of periodontal pathogens. *Passiflora edulis* presents a potential adjunctive therapeutic approach for periodontal disease by mitigating inflammation and bacterial proliferation.

In order to develop improved therapeutic strategies for the prevention and treatment of periodontitis, it is crucial to understand cellular and molecular events. This knowledge is critical for evaluating the potential therapeutic applications of phytochemicals in reducing periodontal damage and promoting tissue regeneration to counteract periodontitis. Thus, the present in vitro study aimed to investigate the modulatory effects of yellow passion fruit bagasse extract (PFBE) on human periodontal cells under microbial stress.

## 2. Materials and Methods

### 2.1. Cell Culture and Treatment

A human immortalized PDL cell line was used for cell culture as previously described [27,28]. The cell line was kindly provided by Dr. Nicolai Miosge, and written consent was obtained according to the ethics regulations of the University of Goettingen (file no.: 27/2/09). PDL cells were maintained in Dulbecco’s Modified Eagle Medium (DMEM+GlutaMAX, Gibco, Invitrogen, Karlsruhe, Germany) supplemented with 10% fetal bovine serum (FBS, Invitrogen), 100 U/mL of penicillin, and 100 μg/mL streptomycin (Invitrogen). Cells were cultured at 37 °C in a humid atmosphere with 5% CO_2_. For experiments, PDL cells were seeded at a density of 1 × 10^5^ cells per well in 6-well plates and grown until reaching 70–80% confluence. Culture medium was refreshed every other day, and FBS concentration was reduced to 1% 24 h before the experiments. To simulate a microbial condition in vitro, *F. nucleatum* ATCC 25586 was used at a concentration previously established in our studies (MOI of 25:1, corresponding to 0.3 × 10^8^ CFU/mL). The bacterial strain was pre-cultured on Schaedler agar plates (Oxoid, Basingstoke, UK) under anaerobic conditions for 48 h. Bacteria were then resuspended in phosphate-buffered saline (PBS, Invitrogen) at a concentration equivalent to 1.2 × 10^9^ bacterial cells/mL and subjected to ultrasonication (160 W for 15 min, repeated twice) to ensure complete bacterial inactivation. Furthermore, PFBE was used for in vitro treatment. The yellow passion fruit (*Passiflora edulis* sp.) is considered a genetic patrimony from Brazil and its use was registered and authorized by the Ministry of Agriculture, Livestock, and Food Supply in the SisGen platform (#A1C93F5). In order to recover polyphenols, the extract was subjected to an extraction process as previously published [29,30]. In summary, the residues of *Passiflora edulis* sp., which are denominated passion fruit bagasse, were acquired, freeze-dried, and ground. Then, the bagasse was subjected to supercritical fluid extraction in order to reduce the fat content. Afterwards, the defatted bagasse was submitted to pressurized liquid extraction to extract the phenolic compounds, such as polyphenols (e.g., piceatannol, scirpusin-B). Three different concentrations (0.25, 0.50, and 1.00 µg/mL) of PFBE were used according to the amount of piceatannol. These concentrations were selected according to an in vitro study in which human keratinocytes were exposed to piceatannol from passion fruit seeds [31]. Cell treatments with *F. nucleatum*, PFBE, or their combinations were performed at 24 h and 48 h. Untreated cells served as control.

### 2.2. Gene Expressions

Total RNA isolation was achieved using the RNeasy Mini Kit (Qiagen, Hilden, Germany) according to the manufacturer’s guidelines. The spectrophotometer NanoDrop ND-2000 (Thermo Fischer Scientific, Waltham, MA, USA) was used to determine the RNA concentration. For cDNA synthesis, five hundred ng of total RNA was reverse transcribed using the iScript Select cDNA Synthesis Kit (Bio-Rad Laboratories, Munich, Germany) as per manufacturer’s instructions. Gene expression levels of tumor necrosis factor-alpha (TNF-α), interleukin (IL)-8, C-C motif chemokine ligand 2 (CCL2), superoxide dismutase (SOD) 2, and matrix metalloproteinase (MMP)-1 were quantified in triplicate by real-time qPCR using the CFX96 Touch Real-Time PCR Detection System (Bio-Rad Laboratories). The reactions were carried out with SYBR green PCR master mix (SsoAdvanced Universal SYBR Green Supermix, Bio-Rad Laboratories) and specific primers (QuantiTect Primer Assay, Qiagen). The reaction mixture contained 12.5 µL master mix, 2.5 µL primer, 9 µL nuclease-free water, and 1 µL of cDNA. The thermal cycling conditions were 95 °C for 5 min, followed by 40 cycles of denaturation at 95 °C for 10 s, and annealing/extension steps at 60 °C for 30 s. Data analysis was performed by using the comparative threshold cycle method.

### 2.3. Protein Levels

The protein levels of TNF-α, IL-8, CCL2, and MMP-1 (DuoSet Human ELISA Kit, DY210, DY208, DY279, DY901B, R&D Systems, Minneapolis, MN, USA), and SOD2 (RayBio Human SOD2 ELISA Kit, ELH-SOD2, RayBiotech, Norcross, GA, USA) were measured in the cell supernatants using commercially available ELISA kits according to the manufacturers’ instructions. The optical density was determined using the multimode microplate reader (BioTek Synergy H1, Agilent, Santa Clara, CA, USA) set to 450 nm. For the ELISA kits from R&D Systems, the readings at 450 nm were subtracted from the readings at 570 nm for optical correction as per manufacturer’s recommendation. Cell numbers were checked and there was no significant difference between groups.

### 2.4. Immunofluorescence

PDL cells were seeded on glass coverslips (Thermo Fisher Scientifics) of 13 mm diameter and placed in 24-well plates (15,000 cells/well). Cells were cultured and treated as described above for up to 60 min. Then, cells were fixed with 4% paraformaldehyde (Sigma-Aldrich, Munich, Germany) at room temperature (RT) for 15 min, washed with PBS, and permeabilized using 0.1% Triton X-100 (Sigma-Aldrich) at RT for 10 min. Subsequently, cells were blocked with a blocking buffer (nonfat dry milk; Bio-Rad Laboratories) at RT for 1 h. After washing, cells were incubated with rabbit anti-nuclear factor-kappa B (NF-κB) p65 primary antibody (1:400; Cell Signaling Technology, Danvers, MA, USA) at RT for 90 min and with CY3-conjugated goat anti-rabbit IgG secondary antibody (1:2000; Abcam, Cambridge, MA, USA) at RT for 45 min. NF-κB nuclear translocation was analyzed with the ZOE Fluorescent Cell Imager (Bio-Rad Laboratories).

### 2.5. Cell Viability Assay

The LIVE/DEAD Viability/Cytotoxicity Kit (Invitrogen) was used as per manufacturer’s recommendations. This assay is based on the simultaneous measurement of live and dead cells through two fluorescent dyes, the calcein AM (green) and the ethidium homodimer-1 (red), respectively, by determining intracellular esterase activity and plasma membrane integrity. For this experiment, PDL cells were grown in 24-well plates (20,000 cells/well) until 70–80% confluence. Then, FBS was reduced to 1% and the cells were treated according to the experimental design for 24 h and 48 h. Untreated cells served as control. After 24 h and 48 h, the medium was removed, and the cells were rinsed twice with 500 µL PBS and incubated with 500 µL of staining solution (2 μM Calcein and 4 μM EthD-1) at RT for 30 min. Next, cells were washed and left with PBS. Then, fluorescence microscopy was performed using ZOE Fluorescent Cell Imager (Bio-Rad Laboratories). Each experiment was repeated three times.

Cell viability was also quantitatively assessed using the alamarBlue kit (Invitrogen) following the manufacturer’s instruction. This assay utilizes a resazurin-based solution that works as an indicator of cell health by measuring the reduction of resazurin to resorufin. Briefly, PDL cells were seeded in flat-bottom 96-well plates at a density of 5000 cells per well and cultured for 24 h. The FBS concentration in the cell culture medium was reduced to 1% for 24 h and then the cells were treated as described above. Untreated cells served as control. Following 24 h and 48 h of treatment, the culture medium was removed and fresh medium containing 10% alamarBlue was added to each well. Cells were then incubated at 37 °C for 4 h. Afterwards, 100 μL of the solubilization solution was transferred to a new 96-well plate and the absorbance was measured using a multimode microplate reader (BioTek Synergy H1, Agilent) at 570 nm and 600 nm wavelengths. Results were expressed as the percentage difference in alamarBlue reduction between the groups using a formula suggested by the manufacturer. Each experiment was repeated three times.

### 2.6. Wound Healing Assay

In order to evaluate the effects of PFBE and *F. nucleatum* on cell migration of PDL cells into the wound area over time, an in vitro wound healing assay was used. PDL cells were seeded into 24-well plates at a density of 50,000 cells per well and allowed to reach confluence over 24 h. A uniform scratch was introduced to each well using the AutoScratch Wound Making Tool (Agilent) according to the manufacturer’s protocol. Briefly, the AutoScratch tool created precise, reproducible wounds across all wells, ensuring consistency in wound width and shape. After scratching, wells were gently washed once with PBS and twice with culture medium to remove detached cell debris and non-adherent cells. Following wound creation, cells were treated with *F. nucleatum* and/or PFBE suspended in fresh culture medium with reduced FBS concentration for 48 h. Untreated cells served as control. The plate was then placed in the incubation chamber of the Lionheart FX Automated Microscope (Agilent) for live imaging. Kinetic high-contrast brightfield images were captured every 2 h over 48 h. The Scratch Assay App (version 1.04, Agilent) was used to automate imaging and analyze wound closure over time. This software automatically provided a detailed report with the percentage of wound closure. Each experiment was repeated three times.

### 2.7. Statistical Analysis

The statistical analysis was accomplished using the software GraphPad Prism version 9.0 (GraphPad Software Inc., San Diego, CA, USA). For quantitative analysis, data were presented as mean values and standard errors of the mean (SEM). Parametric (*t*-test) or non-parametric (Mann–Whitney U test) tests were used. For multiple comparisons, one-way ANOVA with post hoc Dunnett’s test (parametric) or Kruskall–Wallis followed by Dunn’s test (non-parametric) were applied. A significance level of 5% (*p*  <  0.05) was used for all experiments.

## 3. Results

### 3.1. Modulatory Effects of PFBE on Pro-Inflammatory Markers

**Figure 1:**
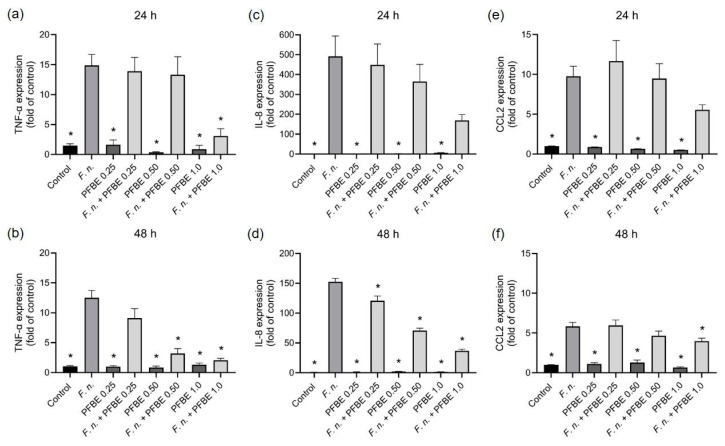
Expressions of TNF-α (**a**,**b**), IL-8 (**c**,**d**), and CCL2 (**e**,**f**) in PDL cells exposed to *F. nucleatum* in the presence and absence of PFBE at three different concentrations of piceatannol (0.25, 0.50, and 1.0 µg/mL) at 24 h and 48 h. Untreated cells served as control. Bars show mean values and SEM. * significant (*p* < 0.05) difference compared with *F. nucleatum* alone.

First, we sought to examine whether PFBE could modulate the stimulatory effects of *F. nucleatum* on the expression of pro-inflammatory markers in PDL cells. As revealed by real-time PCR analysis, *F. nucleatum* significantly upregulated the TNF-α, IL-8, and CCL2 expressions at 24 h and 48 h. PFBE at any concentration had no effect on the expression of these three pro-inflammatory markers. However, the extract was able to counteract the stimulating action of *F. nucleatum* on these three pro-inflammatory markers, especially at high concentrations and over longer periods. For example, the highest PFBE concentration significantly inhibited *F. nucleatum*-induced TNF-α expression at 24 h and the expressions of TNF-α, IL-8, and CCL2 at 48 h (Figure 1a–f).

**Figure 2:**
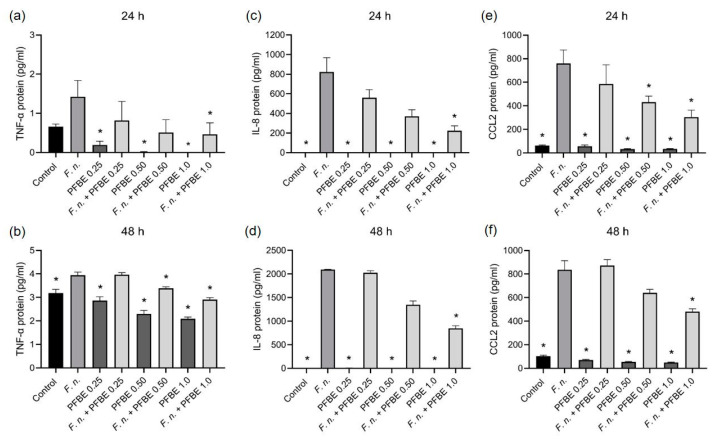
Protein levels of TNF-α (**a**,**b**), IL-8 (**c**,**d**), and CCL2 (**e**,**f**) in PDL cells exposed to *F. nucleatum* in the presence and absence of PFBE at three different concentrations of piceatannol (0.25, 0.50, and 1.0 µg/mL) at 24 h and 48 h. Untreated cells served as control. Bars show mean values and SEM. * significant (*p* < 0.05) difference compared with *F. nucleatum* alone.

Furthermore, we investigated whether PFBE could also modulate the stimulatory effects of *F. nucleatum* on the pro-inflammatory markers at protein level measured by ELISA. *F. nucleatum* significantly increased the protein levels of all markers in cell supernatants at both 24 h and 48 h. Consistent with the transcriptional findings, PFBE alone had no stimulatory effect on the protein levels. The highest concentration of PFBE significantly reduced the stimulative effects of *F. nucleatum* on TNF-α, IL-8, and CCL2 protein levels at both 24 h and 48 h (Figure 2a–f). Additionally, the second highest PFBE concentration significantly reduced the protein levels of TNF-α after 48 h and of CCL2 after 24 h (Figure 2a–f).

### 3.2. Modulatory Effects of PFBE on Markers Involved in Oxidative Stress and Proteolytic Conditions

**Figure 3:**
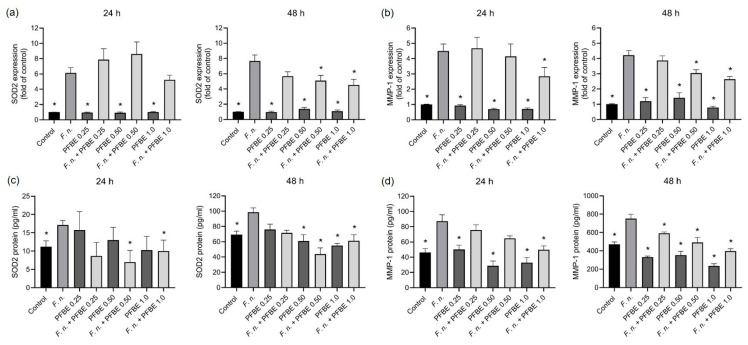
Expressions and protein levels of SOD2 (**a**,**b**) and MMP-1 (**c**,**d**) in PDL cells exposed to *F. nucleatum* in the presence and absence of PFBE at three different concentrations of piceatannol (0.25, 0.50, and 1.0 µg/mL) at 24 h and 48 h. Untreated cells served as control. Bars show mean values and SEM. * significant (*p* < 0.05) difference compared with *F. nucleatum* alone.

We then examined whether PFBE also modulates the effects of *F. nucleatum* on the expression of oxidative stress and proteolytic markers in PDL cells. *F. nucleatum* significantly upregulated the expression of antioxidant enzyme SOD2 and the proteolytic marker MMP-1 at both 24 h and 48 h. In contrast, all concentrations of PFBE alone had no effect. The highest PFBE concentration significantly counteracted *F. nucleatum*-induced MMP-1 expression at 24 h and the expressions of SOD2 and MMP-1 at 48 h (Figure 3a,b). Moreover, the second highest PFBE concentration significantly reduced the SOD2 and MMP-1 expressions induced by *F. nucleatum* at 48 h (Figure 3a,b).

Furthermore, *F. nucleatum* significantly increased the protein levels of both markers in cell supernatants at both 24 h and 48 h. The two highest concentrations of PFBE significantly decreased the stimulatory effect of *F. nucleatum* on SOD2 at both 24 h and 48 h (Figure 3c,d). In addition, the highest concentration of PFBE counteracted *F. nuleatum*-induced MMP-1 protein levels at 24 h, whereas all concentrations of PFBE caused this inhibitory effect at 48 h (Figure 3c,d).

### 3.3. Regulation of F. nucleatum-Induced NF-κB Nuclear Translocation by PFBE

**Figure 4:**
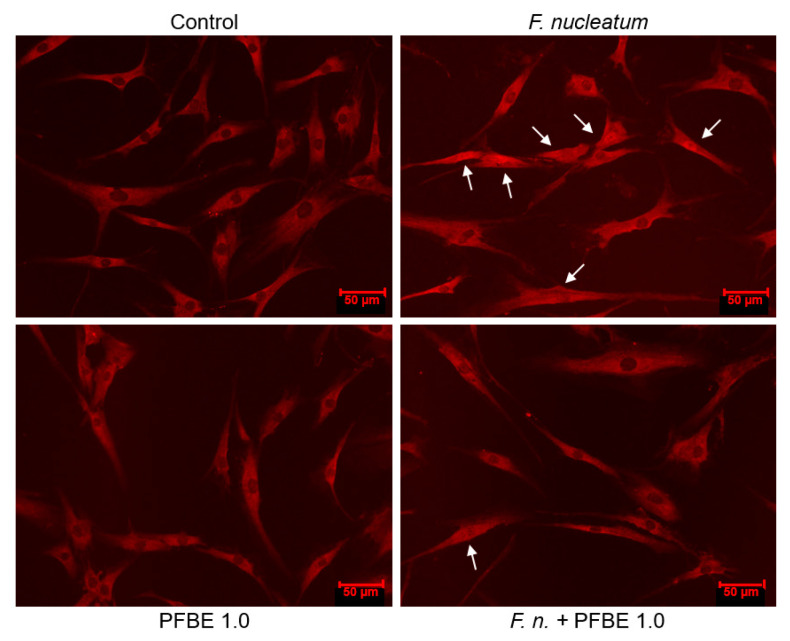
Immunofluorescence analysis of NF-κB nuclear translocation in PDL cells exposed to *F. nucleatum* in the presence and absence of PFBE containing 1.0 µg/mL of piceatannol at 60 min. Untreated cells served as control. Representative images from one experiment are shown. Indicator arrows show NF-κB accumulation within the nucleus of some cells.

As evidenced by immunofluorescence microscopy, *F. nucleatum* caused NF-κB nuclear translocation in some cells at 60 min (Figure 4). We further investigated whether PFBE interferes with the NF-κB pathway triggered by *F. nucleatum*. As shown in Figure 4, PFBE abolished the *F. nucleatum*-stimulated NF-κB nuclear translocation at 60 min.

### 3.4. Effects of F. nucleatum and/or PFBE on Cell Viability

**Figure 5:**
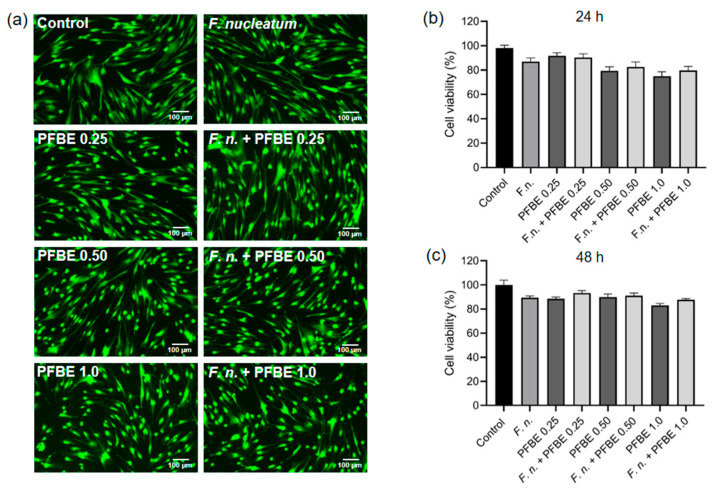
Fluorescence microscopy images of PDL cells exposed to *F. nucleatum* in the presence and absence of PFBE at three different concentrations of piceatannol (0.25, 0.50, and 1.0 µg/mL) at 48 h, as analyzed by live/dead assay. Untreated cells served as control (**a**). Percentage of cell viability of PDL cells exposed to *F. nucleatum* in the presence and absence of PFBE at three different concentrations of piceatannol (0.25, 0.50, and 1.0 µg/mL) at 24 h (**b**) and 48 h (**c**), as analyzed by alamarBlue. Untreated cells served as control. Bars show mean values and SEM. No significant difference was observed between groups.

A live/dead assay was performed to assess possible cytotoxic effects of *F. nucleatum*, PFBE, or their combinations on PDL cells. As shown in Figure 5a, merged images of the “green” (live) and “red” (dead) channels did not demonstrate any red-labeled cells, indicating that all cells exposed to *F. nucleatum* and/or PFBE remained viable. Moreover, cell morphology was also unaffected by *F. nucleatum* and/or PFBE. Cell viability was additionally assessed using the alamarBlue assay, which also showed no significant changes in PDL cell viability at 24 h and 48 h after exposure to PFBE and/or *F. nucleatum* (Figure 5b,c).

### 3.5. Effects of F. nucleatum and/or PFBE on In Vitro Wound Closure

**Figure 6:**
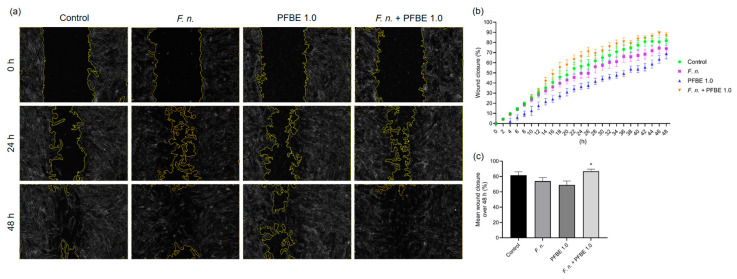
Representative high-contrast brightfield images of in vitro wound closure of PDL cell monolayers exposed to *F. nucleatum* and/or PFBE containing 1.0 µg/mL of piceatannol at 0 h, 24 h, and 48 h (**a**). Percentage of in vitro wound closure in PDL cell monolayers exposed to *F. nucleatum* and/or PFBE containing 1.0 µg/mL of piceatannol over 48 h (**b**). Mean percentage of in vitro wound closure in PDL cell monolayers exposed to *F. nucleatum* and/or PFBE containing 1.0 µg/mL of piceatannol over 48 h (**c**). Untreated cells served as control. * significant (*p* < 0.05) difference compared with *F. nucleatum* alone and PFBE alone.

To assess a possible regulatory role of *F. nucleatum*, PFBE, or their combinations on the wound healing in PDL cell monolayers, an automated in vitro scratch assay was conducted over 48 h. In comparison with the control group, *F. nucleatum* initially showed a more stimulating effect on wound healing, but this was no longer present at later time points. Whereas PFBE alone did not improve wound healing, addition of the extract to *F. nucleatum*-treated cells enhanced the wound closure (Figure 6a–c). As shown in Figure 6c, the mean wound closure percentage after 48 h was also highest with the combination of *F. nucleatum* and PFBE in comparison to *F. nucleatum* alone and PFBE alone.

## 4. Discussion

Periodontal disease is a chronic inflammatory disease affecting the supporting structures of the teeth, including the periodontal ligament and alveolar bone. In recent decades, phytochemical compounds have been extensively investigated as potential therapeutic candidates for periodontitis, leading to a substantial increase in both in vitro and in vivo studies exploring their efficacy, safety, and anti-inflammatory as well as antibacterial properties [32,33]. In the present study, we examined the effects of PFBE on periodontal cells under microbial challenge and observed promising anti-inflammatory and anti-proteolytic activities at both gene and protein levels, along with a potential role in promoting in vitro wound healing. The results of this study suggest that PFBE has the potential to attenuate periodontal inflammation and enhance its resolution. To the best of our knowledge, this is the first study to investigate the beneficial effects of PFBE in a microbial environment in vitro.

PFBE contains a significant amount of bioactive compounds, including piceatannol, scirpusin-B, dicaffeoylquinic acid, citric acid, and (+)-catechin [19,34]. Piceatannol, a hydroxylated analog of resveratrol, is the predominant polyphenol in PFBE [20,21]. The PFBE utilized in our study contained piceatannol at concentrations that had been previously used in an in vitro study in which human keratinocytes were exposed to piceatannol from passion fruit seeds [31].

We evaluated the modulatory effect of PFBE on pro-inflammatory mediators in the presence and absence of *F. nucleatum*. Our results revealed that *F. nucleatum* induced the upregulation of TNF-α, IL-8, and CCL2, key molecules implicated in periodontal inflammation. PFBE reduced the *F. nucleatum*-induced expression of TNF-α, IL-8 and CCL2, i.e., the pro-inflammatory cell response, in a concentration-dependent manner, at both the transcriptional and the protein level. The upregulation of pro-inflammatory cytokines and chemokines is strongly correlated with the initiation and progression of periodontitis, while their inhibition may contribute to regenerative processes in periodontal tissues [35,36,37]. Consistent with our findings, a transgenic adenocarcinoma of mouse prostrate (TRAMP) model has demonstrated that oral administration of piceatannol, derived from PFBE, reduced TNF-α levels in the liver of mice [34]. Furthermore, the anti-inflammatory effects of piceatannol have been reported in various cell lines, including human monocytes (KBM-5), lymphoid cells (Jurkat), breast adenocarcinoma cells (MCF-7), and epithelial cells (HeLa), where piceatannol partially or completely inhibited TNF-α-induced NF-κB activation [38].

There is a lot of evidence that shows a link between oxidative stress and periodontitis. SOD2, a mitochondrial anti-apoptotic and antioxidant enzyme, exhibits an increased expression in gingival tissues of patients with gingivitis and periodontitis compared with healthy individuals [27,39,40]. Its expression decreases with the resolution of gingival and periodontal inflammation [41]. Previous in vitro studies have investigated the presence and function of SOD2 in periodontal cells, revealing that *F. nucleatum* and *P. gingivalis* modulate its expression in oral epithelial cells [42]. Moreover, *F. nucleatum* significantly upregulates SOD2 expression in human PDL cells, while *P. gingivalis* induces its high expression in human gingival keratinocytes [27,43]. In the present study, we also observed a significant upregulation of SOD2 in response to *F. nucleatum*. Interestingly, PFBE significantly reduced the gene expression and protein levels of SOD2 under microbial conditions in our study. Although SOD2 has anti-oxidative and therefore generally protective effects, it can also have negative impacts in chronic diseases in which there is a dysregulation and imbalance of SOD2 [44]. Furthermore, SOD2 exerts anti-apoptotic effects and is a marker of inflammation, as mentioned above. Therefore, the upregulation of SOD2 in periodontitis may contribute to the persistence of the inflammatory cellular infiltrate and, consequently, the progression of this inflammatory disease [27,40]. In our study, PFBE counteracted the *F. nucleatum*-upregulation of SOD2 at transcriptional and protein levels. By these mechanisms, PFBE may have the potential to reduce the number of immunoinflammatory cells and, therefore, periodontal inflammation and destruction.

PFBE also downregulated the *F. nucleatum*-induced gene and protein expression of MMP-1, a key enzyme involved in degrading soft and hard tissues and significantly elevated in periodontitis. The inhibition of MMP-1 synthesis may help preserve the extracellular matrix and mitigate tissue destruction. An anti-inflammatory effect was also observed when piceatannol-pretreated keratinocytes were irradiated with UVB. When fibroblasts were then cultured with the conditioned medium of these piceatannol-pretreated keratinocytes, the MMP-1 protein levels in the supernatants were lower than when the keratinocytes were not subjected to piceatannol pretreatment [31].

In our study, *F. nucleatum* caused NF-κB nuclear translocation in some of the cells and PFBE counteracted this *F. nucleatum*-induced NF-κB nuclear translocation. Interestingly, piceatannol has also reduced the NF-κB activation in other cell types, e.g., HaCaT and adipocytes [45,46]. Other signaling pathways might also have been involved in the anti-inflammatory actions of PFBE, as observed in our study, and further experiments should clarify this aspect.

Although *F. nucleatum* triggered a clear inflammatory response in PDL cells at both transcriptional and protein levels, no significant reduction in cell viability was observed at 24 h and 48 h compared with the control. However, another study was able to demonstrate the negative effects of *F. nucleatum* on cell viability [47]. The differences could be due, among other things, to the use of different bacterial concentrations and preparations or virulence factors, different incubation times, and origins of the PDL cells. It should be emphasized that PFBE had no negative effect on cell viability in our study, which demonstrates the biocompatibility of PFBE at the concentrations tested.

Furthermore, PFBE had a modulating effect on the in vitro wound closure in our study. *F. nucleatum* led to a slight but not significant inhibition of wound closure. Interestingly, PFBE alone also reduced the wound closure, although, again, not significantly. In contrast, when the monolayers were simultaneously exposed to *F. nucleatum* and PFBE, the mean wound closure over 48 h was significantly superior to that in the monolayers treated with *F. nucleatum* or PFBE alone. Piceatannol is known to inhibit wound healing in cancer cells in vitro [22,48]. On the other hand, a combination of bioactive compounds was shown to inhibit the actions of nicotine on PDL and gingival cells, increasing wound healing [49]. Taken together, these data suggest that bioactive compounds can restore and even increase cell migration in environments that have been negatively affected by microbial effects or by smoking.

As in our previous experimental protocols, *F. nucleatum* was used in the form of a lysate, which likely contained lipopolysaccharides as well as other virulence factors, such as proteases, that may have influenced the observed effects in our study. Previous comparative analyses have demonstrated that *F. nucleatum* and the keystone pathogen *P. gingivalis* exert similar effects on periodontal cells [50,51,52]. Furthermore, a significant increase in the production of the pro-inflammatory cytokine TNF-α after exposure to *P. gingivalis* was demonstrated in another cell type (e.g., THP-1 cells). In contrast, the addition of resveratrol—an analogue of piceatannol—significantly decreased TNF-α secretion [53]. However, whether PFBE induces comparable changes in *P. gingivalis*-stimulated PDL cells, as observed with *F. nucleatum* in the present study, remains to be determined in further investigations. We focused on *F. nucleatum* because of its essential role, as a bridging species, in the development and progression of gingivitis and periodontitis. *F. nucleatum* is a Gram-negative, anaerobic, and invasive bacterium that plays a critical role in oral biofilm formation by facilitating the adhesion and coaggregation of other periodontal pathogens, thereby contributing to disease progression [54,55]. Its high prevalence in oral biofilms, both in health and disease, underscores its relevance in periodontal research. Nevertheless, periodontitis is a polymicrobial disease driven by the interplay of multiple bacterial species within a complex biofilm [56]. While the present study focused on *F. nucleatum*, future studies should explore the effects of viable microorganisms and entire periodontal biofilms on these and even other periodontal cells such as gingival fibroblasts, gingival keratinocytes, and bone cells, as this could provide a more comprehensive view of the complexity of periodontal disease and healing.

## 5. Conclusions

In summary, our study revealed that PFBE exerted anti-inflammatory and anti-proteolytic activities on periodontal cells under microbial conditions. The results of the present study suggest that PFBE may have the potential to modulate periodontal inflammation and promote its resolution. Further studies are needed to better understand the underlying molecular mechanisms of the beneficial effects of PFBE and to assess its efficacy in vivo.

## Data Availability

Data used in the present study are available from the corresponding author on reasonable request.

## Abbreviations

The following abbreviations are used in this manuscript:

PDL	Periodontal ligament
PFBE	Passion fruit bagasse extract
DMEM	Dulbecco’s Modified Eagle Medium
FBS	Fetal bovine serum
PBS	Phosphate-buffered saline
RNA	Ribonucleic acid
cDNA	Complementary deoxyribonucleic acid
TNF-α	Tumor necrosis factor-alpha
IL-8	Interleukin-8
CCL2	C-C motif chemokine ligand 2
SOD2	Superoxide dismutase 2
MMP-1	Matrix metalloproteinase-1
PCR	Polymerase chain reaction
ELISA	Enzyme-linked immunosorbent assay
RT	Room temperature
NF-κB	Nuclear factor-kappa B
SEM	Standarde Errors of the means
ANOVA	Analysis of variance
TRAMP	Transgenic adenocarcinoma of mouse prostrate
UVB	Ultraviolet B
